# Time-resolved analysis of *Bacillus subtilis* DB104 Spo0A-mutant transcriptome profile and enhancement of recombinant protein release

**DOI:** 10.71150/jm.2411032

**Published:** 2025-05-27

**Authors:** Ji-Su Jun, Soo Ji Kang, Kwang-Won Hong

**Affiliations:** 1 Department of Food Science and Biotechnology, Dongguk University-Seoul, Goyang-si 10326, Republic of Korea; 2 Synthetic Biology Research Center, Korea Research Institute of Bioscience and Biotechnology (KRIBB), Daejeon, 34141, Republic of Korea; 3 Korean Collection for Type Cultures (KCTC), Biological Resource Center, Korea Research Institute of Bioscience and Biotechnology (KRIBB), Jeongeup 56212, Republic of Korea

**Keywords:** *Bacillus subtilis* DB104, transcriptome analysis, sporulation deficient mutant, recombinant protein, CRISPR-Cas9

## Abstract

Spo0A, the master regulator of sporulation initiation in *Bacillus subtilis*, controls over 500 genes directly or indirectly in early sporulation stages. Although the effects of Spo0A disruption on sporulation have been extensively studied, a comprehensive understanding of the genomic response throughout growth phases remain elusive. Here, we examined the transcriptomic changes in Spo0A mutant strain, R211E, and wild-type across a time-course RNA-seq to identify impacted biological processes and pathways. The R211E strain, which exhibits sporulation deficiency, was constructed using the clustered regularly interspaced short palindromic repeats (CRISPR)-CRISPR associated protein (Cas)9 system, highlighting the critical role of proper Cas9 dosing in gene editing. Functional analysis of 3,010 differentially expressed genes (DEGs) showed significant alterations in sporulation, quorum sensing, metabolism, and biofilm formation. The R211E disrupted the Spo0A-AbrB regulatory pathway, reducing biofilm formation and enhancing flagellar gene expression. Up-regulated metabolic pathways, including glycolysis, histidine, and purine biosynthesis, increased cell numbers during vegetative growth. Further, the mutant displayed elevated vegetative autolysin expression, resulting in reduced cell viability in the stationary phase. We also introduce the novel potential of R211E in a recombinant protein expression system that facilitated protein release into the supernatant, providing valuable insight for future research in metabolic engineering and efficient production systems in *B. subtilis*.

## Introduction

*Bacillus subtilis*, the most extensively studied model of Gram-positive bacteria, has been widely used in basic genetic research and as a host for recombinant protein expression (Kobayashi and Ogasawara, 2002; Zhang et al., 2020). Upon nutrient deprivation, *B. subtilis* can produce dormant endospores that endure extreme conditions such as heat, UV radiation, and chemical solvents (Setlow, 2006). Sporulation begins with an asymmetric cell division; the smaller forespore is engulfed by the larger mother cell (McKenney et al., 2013). The mother cell then nurtures the development of the forespore into a mature spore, eventually lysing to release it (Smith and Foster, 1995).

The sporulation process is initiated by the DNA-binding protein Spo0A, a transcription factor that directly activates over 500 genes in the early and middle stages of sporulation (Fawcett et al., 2000). Spo0A’s activity is regulated through phosphorylation-mediated signal transduction, led by five histidine kinases (Jiang et al., 2000). Phosphoryl groups are transferred from the kinase to Spo0A via Spo0B and Spo0F, resulting in the activated form of Spo0A, Spo0A~P (Perego et al., 1989). Accumulated Spo0A~P then triggers the expression of sporulation genes and facilitates polar septation, leading to the formation of a forespore and mother cell compartment within the cell (Fujita and Losick, 2005). Subsequently, sigma factors orchestrate morphological changes that culminate in the formation of a mature spore through temporal and spatial regulation (Steil et al., 2005). The forespore-specific sigma factor σ^F^ activates and directs the expression of forespore-specific genes, including the late-stage forespore-sigma factor σ^G^. Conversely, σ^E^, activated in the mother cell, drives the transcription of mother cell-specific genes, including the late-stage mother cell-sigma factor σ^K^.

In the food industry, the clustered regularly interspaced short palindromic repeats (CRISPR)-CRISPR-associated protein (Cas)9 gene editing system, which remains its food-grade status and prevents foreign DNA incorporation into the genome, has been recently employed (So et al., 2017; Zhou et al., 2023). Additionally, it is crucial to produce food-related materials using microbial cells, minimizing the risk of spore formation. Several studies have detailed various strategies to inhibit sporulation in spore-forming bacteria and *B. subtilis* (Eswaramoorthy and Fujita, 2010; Ireton et al., 1994; Wang et al., 2007; Zhou et al., 2019). Specifically, traits of *B. subtilis* lacking Spo0A have been previously researched in terms of phenotype and genotype (Hatt and Youngman, 1998; Pedrido et al., 2013; Wang et al., 2020). These cells cannot form spores, show reduced biofilm development, and have an increased susceptibility to lysis (Hamon and Lazazzera, 2001; Kodama et al., 2007). However, most of these studies have concentrated on specific phenomena, leaving gaps in our understanding of the broader transcriptomic changes and metabolic responses associated with the absence of this key regulator. Evaluating the impact of a Spo0A mutation on overall metabolism during growth is therefore essential.

Next-generation sequencing (NGS) offers comprehensive and precise analysis of gene expression. RNA-sequencing (RNA-seq), a widely used NGS-based technology, enables simultaneous exploration of thousands of genes. Unlike microarray technology, RNA-seq does not require prior knowledge of the reference transcriptome, suffers minimal background signal, provides a broader dynamic range of expression levels, and requires only a small amount of total RNA for quantification (Kogenaru et al., 2012; Mantione et al., 2014). Consequently, RNA-seq has recently been utilized for gene expression profiling (Fishman et al., 2024; Valdés-Hernández et al., 2023; Yu et al., 2024).

In this study, we examine the effects of loss-of -function mutation of Spo0A on the transcriptome of exponentially growing cultures. We compared the transcriptome of a Spo0A mutant (R211E) to that of the wild-type strain using RNA-seq. The R211E strain, engineered via CRISPR-Cas9, exhibited a sporulation deficiency. We analyzed the changes in genes, biological processes, and pathways through functional analysis of differentially expressed genes (DEGs), gene ontology (GO) enrichment, and KEGG pathway analysis. Furthermore, the effects of Spo0A function loss on protein expression were investigated using enhanced green fluorescent protein (eGFP) as a reporter. These findings offer insight into the molecular mechanisms of Spo0A’s regulatory functions and its inactivation’s potential application in recombinant protein production.

## Materials and Methods

### Bacterial strains, growth conditions, and transformation

Table 1 lists the bacterial strains and plasmids used in this study. *Escherichia coli* DH5α was employed for the construction of recombinant plasmids. The transformation of *E. coli* was performed using the heat shock method (Froger and Hall, 2007). *B. subtilis* DB104 (WT) and *B. subtilis* DB104 Spo0A R211E (R211E) were transformed as previously described (Vojcic et al., 2012). For the transformation, approximately 200 ng and 2 mg of plasmid DNA were used for WT and R211E, respectively. The strains were cultured in lysogeny broth (LB) medium (1% tryptone, 0.5% yeast extract, and 1% NaCl) or on LB agar plates at 37℃. If needed, the medium was supplemented with ampicillin (50 μg/ml), chloramphenicol (25 μg/ml) or kanamycin (10 μg/ml).

### Plasmid construction

**Construction of pUB19-Cas90A and pUB19L-Cas90A:** Oligonucleotides used in this study are listed in Table S1. The construction scheme for pUB19L-Cas90A is briefly described in Fig. S1. To express the Cas9 protein in *Bacillus*, the promoter of *cas9* was replaced by the promoter of *amyE* (P_amyE_) from *B. subtilis* DB104. The promoter region upstream of *amyE* (413 bp) was amplified using polymerase chain reaction (PCR) with chromosomal DNA from *B. subtilis* DB104 as the template and the primer pair amyE-F/amyE-R. The pCas9 (Addgene, #42876) backbone, excluding the original *cas9* promoter, was amplified with the primer pair pCas9-F/pCas9-R. These two PCR products were digested with SpeI and XhoI, then ligated to form the pP_amyE_-Cas9 plasmid. To make point mutation in *spo0A*, optimal gRNA sequences were designed using CRISPR gRNA design tool CHOPCHOP (https://chopchop.cbu.uib.no/, Labun et al., 2019). Oligonucleotides for the 20 bp gRNA (0A-F and 0A-R) were synthesized and inserted at the BsaI site in pP_amyE_-Cas9 (Fig. S1B). The resulting plasmid that contained *spo0A*-targeting gRNA and P_amyE_ was designated pP_amyE_-Cas90A (Fig. S1A). The pP_amyE_-Cas90A plasmid served as a template for amplifying tracrRNA-Cas9-crRNA fragments using the CRISPR-F and CRISPR-R primers; this was followed by digestion with PstI and BglⅡ. This fragment was then ligated with BamHI/PstI-digested *E. coli*-*Bacillus* shuttle vector pUB19 (Kang et al., 2019), resulting in pUB19-Cas90A (Fig. S1A). To introduce a low-copy-number mutation within the pUB19-Cas90A replicon, a G → T conversion occurred at position 7 in the *repB* gene sequence (Leonhardt, 1990). A fragment containing a single nucleotide modification was generated from the pUB19-Cas90A plasmid by overlap PCR of two amplified products using the primer pairs LC-F/LC-OR and LC-OF/LC-R. Subsequently, this fragment was inserted into the pUB19-Cas90A at the PstI and BglⅡ sites to produce pUB19L-Cas90A (Fig. S1A).

**Construction of pUC19-R0A:** To construct pUC19-R0A for a point mutation in *spo0A*, the 257 bp N-terminal region upstream and the 207 bp C-terminal region downstream of the target were amplified from *B. subtilis* DB104 chromosome DNA using primer sets HDR-F/HDR-OR and HDR-OF/HDR-R, respectively. Subsequent fusion PCR generated a 430 bp donor DNA template with substituted sequences of *spo0A*, Arg211 (CGT) to Glu211 (GAA). The donor DNA and pUC19 were then digested with BamHI and HindⅢ, and ligation resulted in pUC19-R0A (Fig. S2).

**Construction of protein expression vectors:** Protein expression was analyzed using eGFP as a reporter protein. Primers for constructing the protein expression vector are listed in Table S1. Promoter sequences were amplified from *B. subtilis* DB104 chromosomal DNA. The pUB19 vector skeleton was created by digesting pUB19-P_sdp_-egfp-2 (Jun et al., 2023a) with MluI and HindⅢ. To construct pUB19-P_hag_-egfp, the *hag* promoter (P_hag_) with a strong Shine Dalgarno (SD) sequence (TAAGGAGG) was amplified using the primer set hag-F/hag-OR. The *egfp* gene was amplified from pUB19-P_sdp_-egfp-2 using the primer set hag-OF/eGFP-R. These two PCR fragments were fused by overlap extension PCR and subcloned into the pUB19 vector. Meanwhile, the *ssrA* promoter (P_ssrA_) was amplified using the primer set ssrA-F/ssrA-OR. Given that *ssrA* codes for the transfer-messenger RNA (tmRNA) gene, the SD sequence from the *sdp* operon was utilized. The *egfp* gene with this SD sequence was amplified from pUB19-P_sdp_-egfp-2 using the primer set *ssrA*-OF/eGFP-R. The PCR products were then recombined and inserted into the pUB19 vector, resulting in pUB19-P_ssrA_-egfp.

### CRISPR-Cas9-mediated gene editing of *B. subtilis* DB104

*B. subtilis* DB104 was transformed with Cas9-carrying plasmid (pUB19-Cas90A and pUB19L-Cas90A) and a donor DNA-carrying plasmid (pUC19-R0A). Competent cells were prepared using a previously described method with several modifications (Vojcic et al., 2012). Briefly, a single colony was inoculated into starvation medium 1 (SM1, 0.2% ammonium sulfate, 1.4% dipotassium hydrogen phosphate, 0.6% potassium dihydrogen phosphate, 0.07% sodium citrate, 0.5% glucose, 0.02% magnesium sulfate, 0.2% yeast extract, and 0.025% casamino acids) and was cultured overnight at 37℃ under aeration with shaking at 250 rpm. The overnight culture was transferred to SM1 medium (initial OD_600_ = 0.5) and incubated for 3 h. An equal volume of starvation medium 2 (SM2, 0.2% ammonium sulfate, 1.4% dipotassium hydrogen phosphate, 0.6% potassium dihydrogen phosphate, 0.07% sodium citrate, 0.5% glucose, 0.08% magnesium sulfate, 0.1% yeast extract, 0.01% casamino acids, and 0.05% calcium chloride) was then added to the cell culture and histidine was supplemented to final concentration of 200 μg/ml. After 2 h of incubation, 200 ng of the Cas9-carrying plasmid and 1 μg of the donor DNA-carrying plasmid were added to 500 μl of competent cells and incubated for 30 min. To recover the cells, 300 μl of fresh LB medium was added and the cells were cultured for an additional 30 min. The transformed cells were spread on LB agar plate containing kanamycin. Colonies grown on selective plates were tested for the correct mutations. The obtained colonies were evaluated for editing confirmation through phenotypic screening and DNA sequencing. The confirmed mutants were sub-cultured in LB without antibiotics for three consecutive days. Dilutions of the culture (10^-3^–10^-5^) were spread on LB plates to obtain single colonies. The plasmid-cured mutants were identified by drug-sensitive colonies. Sporulation efficiency was verified by determining the number of total cells and endospores. An aliquot of the culture was centrifuged, resuspended in an equal volume of 1.5 mg/ml lysozyme, and incubated at 37℃ for 10 min to remove viable cells. The cultures, both with and without lysozyme treatment, were spread onto LB plates. Sporulation efficiency was confirmed by comparing the colony forming unit (CFU)/ml of the spores to the CFU/ml of the total at 36 h.

### Growth curve

A single colony of WT or R211E strain was inoculated into 5 ml of LB broth and incubated at 30℃ overnight. The culture was shaken at 250 rpm. Subsequently, the overnight culture was transferred into 50 ml of fresh LB broth in 500 ml baffled flasks with an initial OD_600_ = 0.1 and incubated under identical conditions. At various time points, samples were taken, and cell density was measured using the OD_600_ on a spectrometer. All experiments were performed in triplicate.

### RNA extraction, cDNA library preparation, and RNA sequencing

RNA was extracted from the R211E culture samples at specified time points (8, 10, 12, and 15 h) as described previously (Jun et al., 2023b). The RNA, derived from three biological replicates with one used for RNA-seq, was processed. RNA-sequencing was carried out by Macrogen (Korea) on an Illumina NovaSeq 6000 using a TruSeq Stranded Total RNA Library Prep Gold Kit (Illumina, USA). Sequencing quality was assessed using FastQC v0.11.7 (http://www.bioinformatics.babraham.ac.uk/projects/fastqc/). To eliminate adapter sequences and low-quality bases, Trimmomatic 0.38 (http://www.usadellab.org/cms/?page=trimmomatic) was utilized. Cleaned reads were then mapped to the *B. subtilis* subsp. *subtilis* str. 168 genome (GenBank accession number: AL009126.3) using HTseq version 0.10.0 and Bowtie 1.1.2. All raw transcriptome data were deposited in the NCBI Sequence Read Archive (https://ncbi.nlm.nih.gov/sra) database under the accession number PRJNA1162568. Our previous transcriptome data of *B. subtilis* DB104 (WT), the parental strain of R211E, were used for control in this study and are publicly available in the NCBI SRA database (accession number PRJNA934277) (Jun et al., 2023b). The data were obtained using the same methods except for the library kit.

### Functional annotation and DEG analysis

For differential expression analysis, a trimmed Mean of M-values (TMM) normalization were performed using the EdgeR Software (version 3.16.5) (Robinson et al., 2010; Robinson and Oshlack, 2010). DEGs were identified by calculating the transcripts per kilobase million (TPM) value (Anjum et al., 2016). DEGs were defined as genes with a *p*-value < 0.05 and |fold-change| ≥ 2. Gene Ontology (GO) enrichment analysis was conducted using Cytoscape BiNGO (Version 3.10.1) (Maere et al., 2005) based on *p*-value < 0.03 and a Benjamini–Hochberg-corrected *p*-value < 0.03. DEGs were annotated as either up-regulated or down-regulated. KEGG pathway analysis was conducted to explore pathways altered in the two strains.

### Real-Time quantitative PCR (RT-qPCR)

To confirm the accuracy of RNA-seq results, the expression of 8 DEGs was analyzed by RT-qPCR across three biological replicates. RNA-seq analysis was conducted on one of the three replicated samples, with the remaining two examined collectively to ensure experimental consistency. A total of 8 DEGs that exhibit the extreme fold changes were selected for validation via RT-qPCR. One μg of RNA was used to synthesize cDNA using random hexamers (Roche, Switzerland) and M-MLV reverse transcriptase (Promega, USA), following the manufacturer’s protocol. RT-qPCR was performed using Taq Pro Universal SYBR qPCR Master Mix (Vazyme, China) on a CFX Connect Real-Time System (Bio-Rad, USA). Gene-specific primers are listed in Table S3. Each sample was analyzed in triplicate. To normalize the target gene’s relative expression levels, *rpsJ* served as the internal reference gene. The relative expressions of the selected genes were calculated using the 2^−∆∆Ct^ method (Livak and Schmittgen, 2001).

### Measurement of fluorescence intensity of enhanced eGFP

A colony of *B. subtilis* carrying an expression vector was inoculated into 10 ml of LB broth containing 10 μg/ml kanamycin and incubated at 30℃ with shaking at 250 rpm. This preculture was then transferred to 50 ml of fresh LB broth containing the same antibiotics in a 500 ml baffled flask, starting at an OD_600_ of 0.1. After 48 h of cultivation, 100 μl of the sample was placed into a black flat 96-well plate (SPL Life Sciences, Korea). The fluorescence intensity of eGFP was measured using a Victor^TM^ X4 multi-plate reader (PerkinElmer, USA). The activity of eGFP was characterized by the ratio of fluorescence intensity of eGFP to dry cell weight (DCW). To measure DCW, 1 ml aliquots of culture were transferred to preweighed 1.5 ml microtubes, centrifuged for 5 min at 13,000 × g, and supernatants were discarded. The cell pellets were then dried at 80℃ until a constant weight was achieved, typically after 24 h. The dry cell mass was calculated from the final weights. Data represent the average from three independent experiments, and statistical significance was assessed using one-way analysis of variance (ANOVA), followed by Duncan’s post hoc test using IBM SPSS Statistics 27.0 software (SPSS Inc., USA). Differences with P < 0.05 were considered statistically significant.

## Results

### Construction of sporulation-deficient strain

**Validation of CRISPR-Cas9 function in *B. subtilis* DB104:** pCas9 is a *Streptococcus pyogenes* CRISPR-Cas9 system based-plasmid, which can facilitate gene editing in *E. coli*. To develop a gene editing system in *B. subtilis*, the CRISPR-Cas9 components from pCas9 were transferred to the *E coli*-*Bacillus* shuttle vector pUB19, with the *cas9* promoter replaced by P_amyE_, resulting in pUB19-Cas90A (Fig. S1A). However, no transformants were achieved when pUB19-Cas90A and pUC19-R0A were co-transformed into *B. subtilis* DB104 in 10 separate attempts. We hypothesized that pUB19-Cas90A might cause excessively high Cas9 expression levels, obstructing cellular repair mechanisms. Consequently, we aimed to decrease Cas9 expression by reducing the copy number of the pUB19 vector. To achieve this, the replicon *repB* gene sequence was modified by substituting G with T at position 7 (Fig. S1A), referencing the report by Leonhardt (1990). As a result, 21 colonies were obtained from the co-transformation of pUB19L-Cas90A and pUC19-R0A, with six colonies randomly selected for *spo0A* mutant verification.

**Fig. 1.**
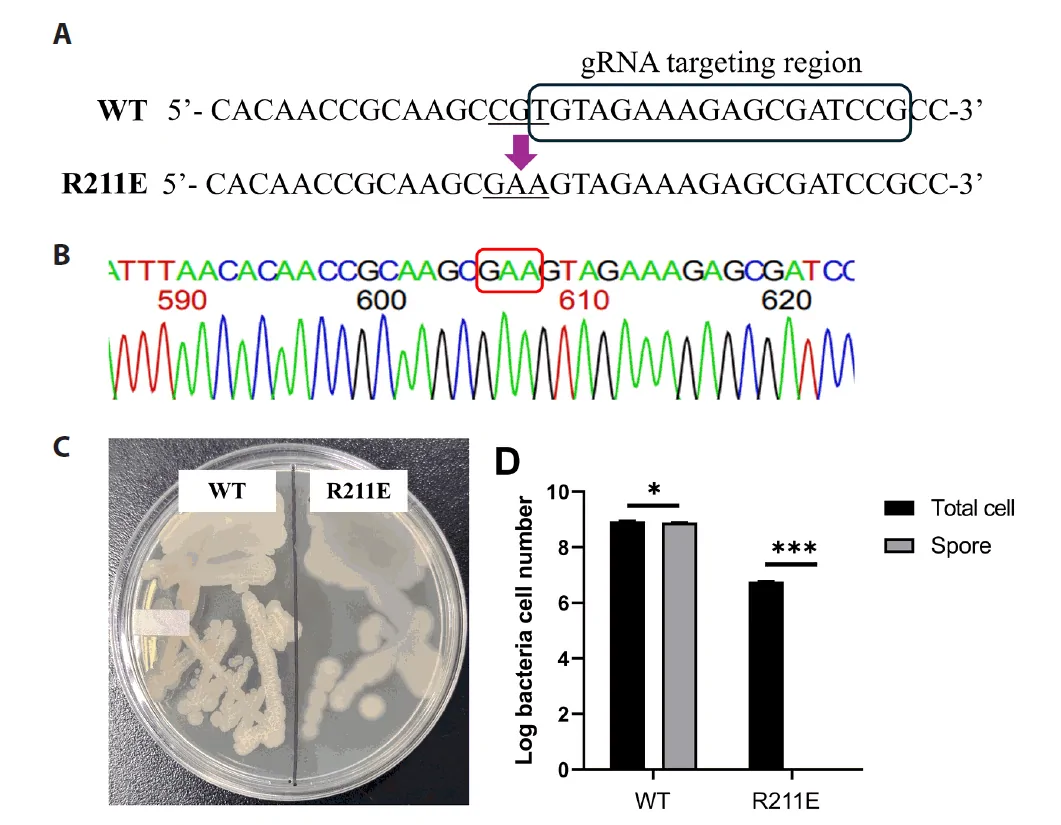
Confirmation of the mutation of *spo0A*. (A) The gRNA-targeted region is boxed. The changed nucleotides are underlined. The three-nucleotide mutation changed the 211^th^ arginine to glutamic acid. (B) The DNA sequencing result of R211E. (C) The morphology of R211E (right) became more transparent compared to WT (left). (D) Sporulation efficiency in R211E and WT, with means and standard deviations from three independent replicates presented. Significant levels are indicated by “*ns*” and “***” for *p* < 0.001 and *p* > 0.05, respectively (Student’s t-test).

**Loss-of-function mutation of Spo0A using the CRISPR-Cas9 system:** Six colonies were randomly selected and evaluated through morphological assessments and genomic DNA sequencing. The *spo0A* mutation is depicted in Fig. 1A. Consequently, 83.3% (5 out of 6) of the colonies displayed a transparent phenotype and harbored the *spo0A* mutation (Fig. 1B and 1C). Additionally, the plasmid curing efficiency of pUB19L-Cas0A was 65%, indicating that plasmids were readily eliminated through serial culture in antibiotic-free conditions. The strains resulting from the *spo0A* mutation, referred to as R211E, exhibited no spores after 36 h of incubation, whereas 96% of WT cells formed spores at the same time (Fig. 1D). This result demonstrates that the single amino acid substitution in Spo0A effectively resulted in a sporulation-deficient phenotype.

### Cell growth

**Fig. 2.**
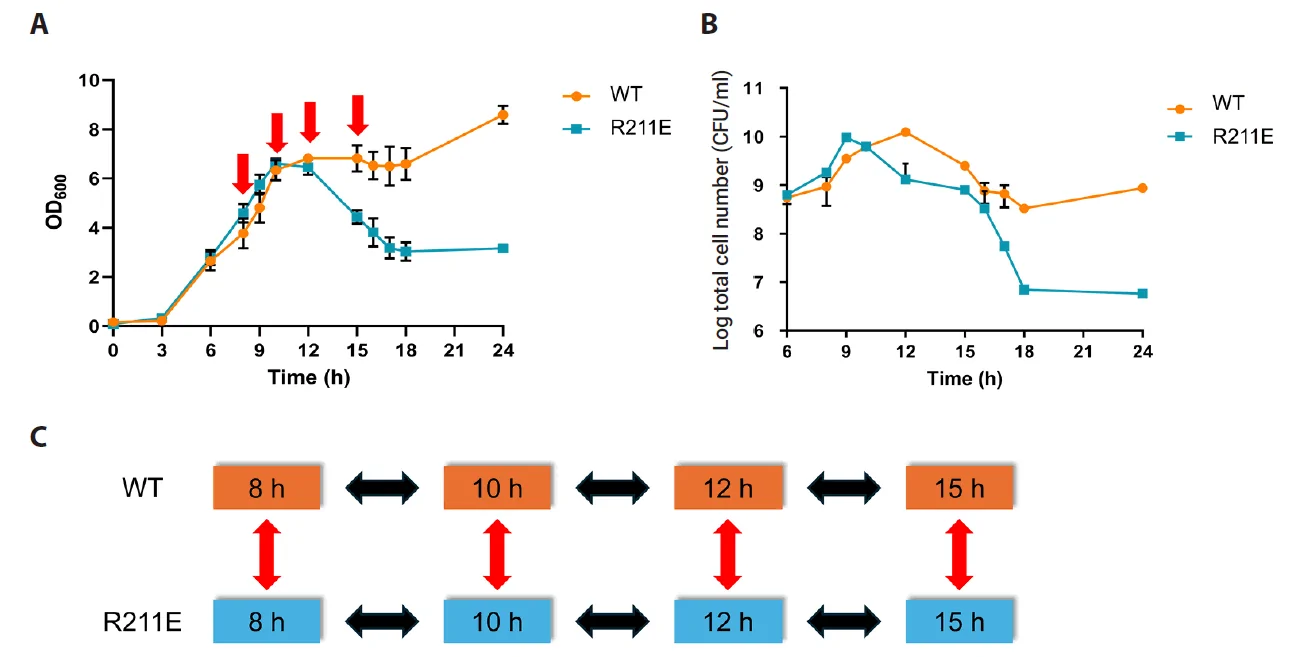
Growth curves as a plot of optical density. (A) Optical density (OD_600_) and (B) cell number for *B. subtilis* DB104 (WT) and *B. subtilis* DB104 Spo0A R211E (R211E). Total RNAs were extracted at times indicated by arrows. (C) Pairwise comparisons between time points within each strain (black arrow) and between strains at each time point (red arrow) were conducted in this study.

The growth of R211E cells was compared to its parental strain, WT, by monitoring cell density using optical density measurements at OD_600_ (Fig. 2A). No noticeable differences were observed in the cell density of the two strains until 12 h when they finished the exponential growth. After the first 12 h, R211E exhibited a significant decrease in optical density, unlike the continuous increase noted in WT. Viable cell numbers at various times were also determined by plate counting (Fig. 2B). WT total cell counts were 9.3 × 10^8^, 6.1 × 10^9^, 1.2 × 10^10^, and 2.5 × 10^9^ CFU/ml at 8, 10, 12, and 15 h, respectively. For R211E, the counts were 1.8 × 10^9^, 6.2 × 10^9^, 1.2 × 10^9^, and 8.0 × 10^8^ CFU/ml at the same respective hours. At 9 h, R211E had a 1.9-fold higher cell number, yet it displayed lower cell numbers than WT after 12 h.

### RNA sequencing and analysis of DEGs

Cell samples from R211E were collected at four-time points based on the growth curve: the middle of exponential growth (8 h), the end of exponential growth (10 h), the beginning of stationary phase (12 h), and the middle of stationary phase (15 h) (Fig. 2A). Total RNAs were extracted at each time point for RNA-seq. We utilized WT transcriptome dataset from a previous study (Jun et al., 2023b), available for download from the NCBI SRA database using accession number PRJNA934277. The sequencing data were likewise obtained using RNA samples from the same time points. The quality control results are available in Table S2. Sequencing yielded between 36.5 and 21.9 million reads per sample. After trimming low-quality reads, clean reads ranged from 36.0 to 21.5 million, accounting for over 97.7% of the raw reads. Additionally, 86.8% to 95.8% of these reads could be mapped successfully to the reference genome (GenBank accession number: AL009126.3), with more than 95% exhibiting a quality score above 30 across all samples. These results confirm the high quality of the sequencing data, suitable for subsequent analyses.

**Fig. 3.**
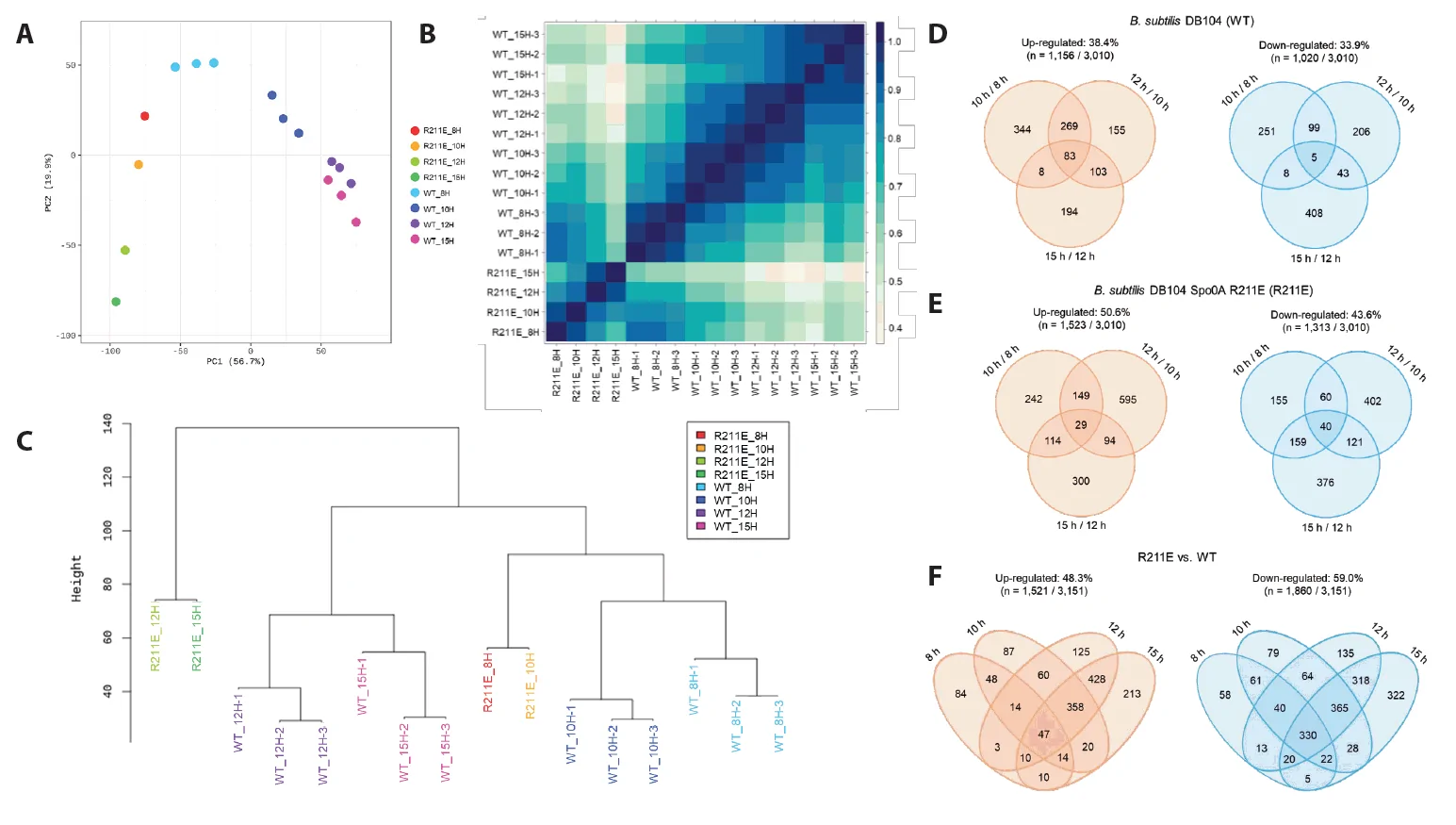
Analysis of the RNA-seq data. (A) Principal component analysis and (B) Pearson’s correlation analysis of all samples. (C) Hierarchical clustering of the samples. The number of up- and down-regulated DEGs in pairwise comparison between time points in (D) WT and (E) R211E. (F) The number of up- and down-regulated DEGs in pairwise comparison between strains at each time point.

Differences among samples were confirmed through principal component analysis and Pearson’s correlation coefficient analysis (Fig. 3A and 3B). Furthermore, WT exhibited high Pearson’s correlation coefficients, affirming the reproducibility among three biological replicates. The similarity in gene expression among samples was also evident in hierarchical clustering (Fig. 3C). For instance, gene expression between two strains, WT and R211E, closely clustered during the exponential growth. However, gene expression in R211E during the stationary phase was significantly different from other samples, whereas in WT, it clustered relatively closer to samples from earlier time points. These results suggest that gene expression changes are triggered at the beginning of stationary phase in R211E.

Genes with a *p*-value < 0.05 and |fold change| ≥ 2 were identified as DEGs (see volcano plots in Fig. S3). As a result, a total of 3,010 DEGs were identified within each strain comparison (Fig. 2C, black arrow). Within WT, 1,156 (38.4%) and 1,020 (33.9%) genes were up-regulated and down-regulated, respectively (Fig. 3D). Notably, 83 genes were consistently up-regulated and 5 genes were consistently down-regulated across all compared groups. In R211E, 1,523 (50.6%) and 1,313 (43.6%) genes were up-regulated and down-regulated, respectively (Fig. 3E). Additionally, 3,151 DEGs were identified in time-point-pairwise comparisons between strains (Fig. 2C, red arrow). Of these, 1,521 (48.3%) were up-regulated and 1,860 (59.0%) were down-regulated, with 47 and 330 genes consistently up- and down-regulated at all times, respectively (Fig. 3F).

### The DEGs with top up-regulation and down-regulation

The top 10 up- and down-regulated genes with significant fold-change values were validated, as listed in Table 2. Genes in the *hisZGDBHAFI* operon involved in histidine biosynthesis were among the most up-regulated (in R211E, from the middle to end of exponential growth). In contrast, the top 10 down-regulated DEGs, identified by their fold-change values, were mostly associated with sporulation and predominantly observed in the comparisons between two strains at the beginning or middle of stationary phase. Moreover, these down-regulated genes exhibited much higher fold-change values than the up-regulated genes, indicating strong repression of sporulation in R211E.

### Validation of RNA-seq data by RT-qPCR

To validate the RNA-seq results, the expression of eight genes from Table 2 was analyzed using RT-qPCR (Fig. S4). RNA-seq analysis was conducted using one of the three replicated samples, and the other two samples were analyzed jointly to confirm experimental reproducibility among biological replicates. A comparison of fold changes in gene expression evaluated the correlation between RNA-seq data and RT-qPCR. The RT-qPCR results confirmed that the expression pattern of these genes was consistent with the RNA-seq results and demonstrated experimental reproducibility.

### Functional analysis of DEGs

To assess the biological differences in genes across each time and strain pairwise comparison (Fig. 2C), GO enrichment analysis was conducted. For each comparison pair, the set of up-regulated and down-regulated DEGs were investigated separately. A total of 384 biological process (BP) terms were found to be significantly over-represented (*p*-value < 0.03 and Benjamini–Hochberg-corrected *p*-value < 0.03). All enriched GO terms in BP are listed in Table S4. To explore the pathways influenced by the Spo0A mutation, KEGG pathway analysis was performed on the DEGs between strains at each time point. The DEGs were enriched in 84 pathways (false discovery rate [FDR] < 0.5) and they are enumerated in Table S5. To make the results more concise, the findings from both analyses were categorized as follows.

**Fig. 4.**
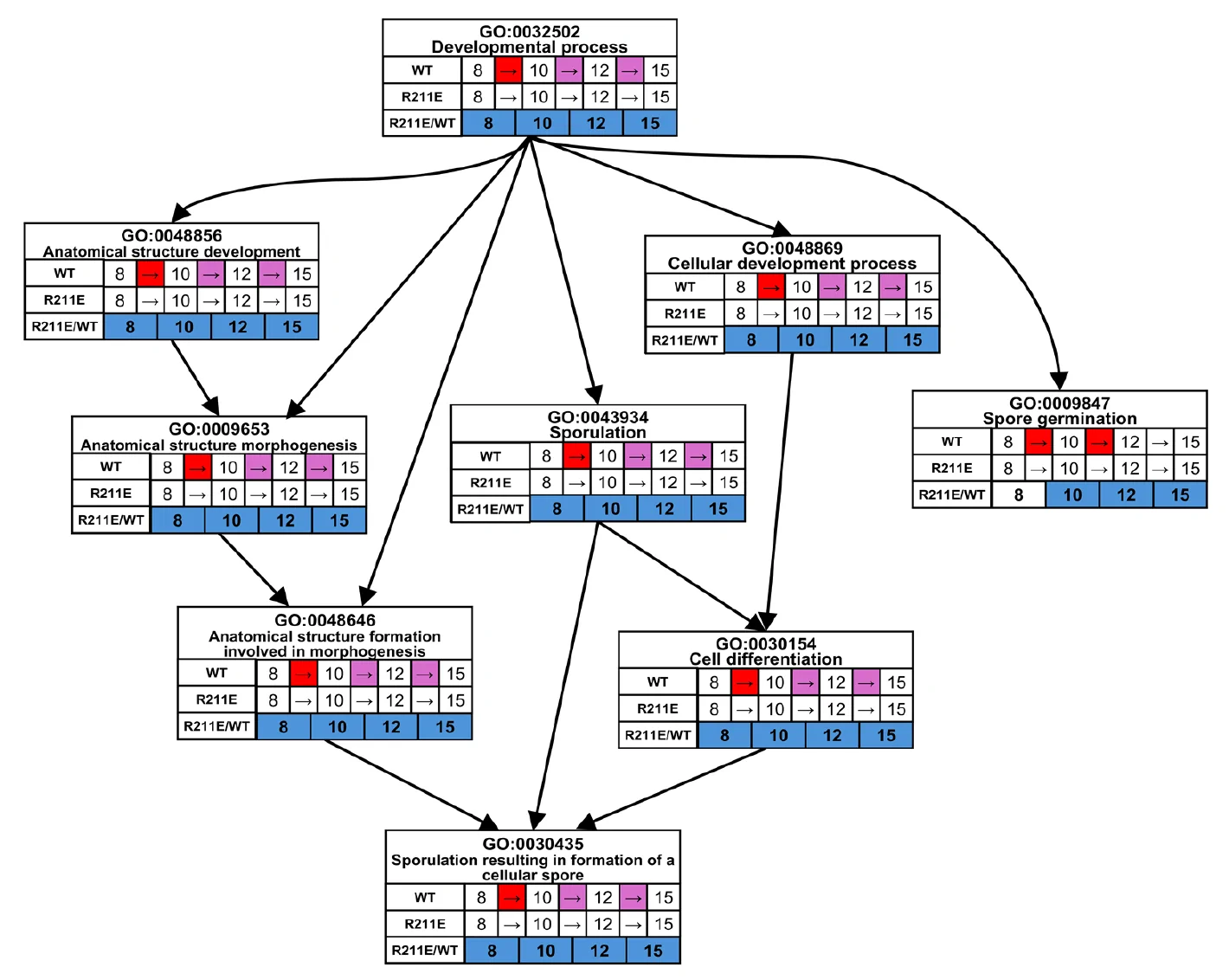
Enriched GO terms associated with sporulation and germination. The directed acyclic hierarchical graph (DAG) visualizes networks of identified GO terms related to sporulation and germination. In each comparison, the colors red and blue denote up- and down-regulation, respectively, while pink signifies enrichment in both up- and down-regulated DEG sets.

**Fig. 5.**
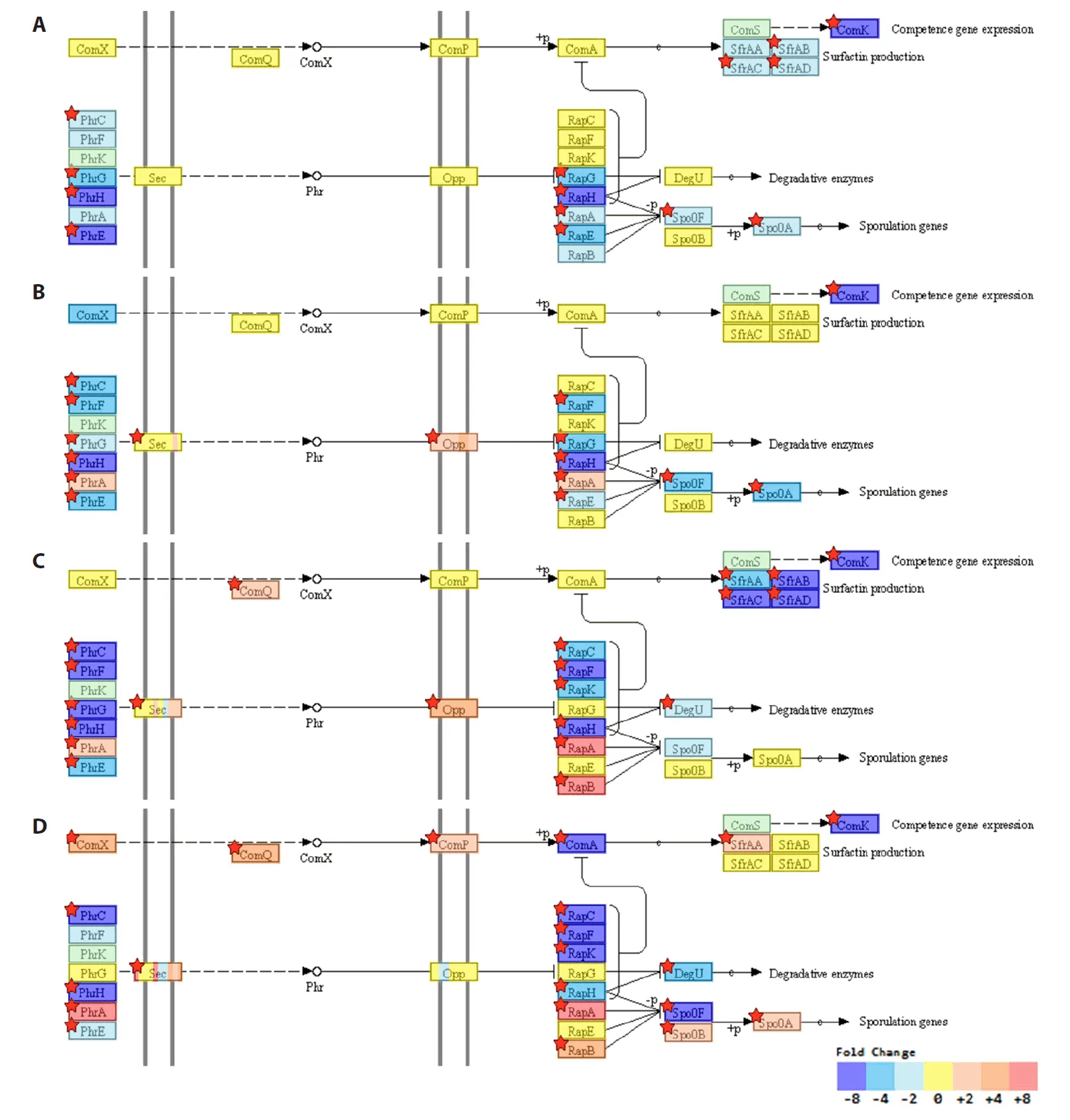
KEGG pathway enrichment of quorum sensing. Quorum sensing pathway highlights differentially expressed genes in R211E versus WT at (A) the middle of exponential growth (8 h), (B) the end of exponential growth (10 h), (C) the beginning of stationary phase (12 h), and (D) the middle of stationary phase (15 h). Fold change values are indicated by a color key. Colors blue, red, and yellow colors represent down-regulated, up-regulated, and non-differentially expressed genes, respectively. Green indicates genes that are not mapped. Genes marked with a red star are considered significantly different.

**Sporulation and germination:** The developmental process (GO:0032502) was significantly down-regulated in R211E compared to the WT at distinct time points (Fig. 4). Alongside the sporulation rate, child terms related to sporulation (GO:0043934), such as anatomical structure development (GO:0048856), cell differentiation (GO:0030154), and sporulation resulting in the formation of a cellular spore (GO:0030435), were consistently down-regulated in R211E. Additionally, the data indicate that sporulation-related terms were up-regulated in WT during the transition from the middle to the end of exponential growth (8 h → 10 h), from end of exponential growth to the beginning of the stationary phase (10 h → 12 h), and from the beginning to the middle of stationary phase (12 h → 15 h). At these later timepoints (10 h → 12 h and 12 h → 15 h), they were also enriched in the down-regulated DEG set. Conversely, in R211E, these terms were not significantly enriched in any timepoint comparisons, suggesting that sporulation related genes were turned off from the exponential growth. A similar pattern was observed with the term spore germination (GO:0009847), which was also down-regulated in R211E. The KEGG pathway analysis revealed significant enrichment in the quorum sensing (bsu02024) pathway, closely related to sporulation (Fig. 5).

**Fig. 6.**
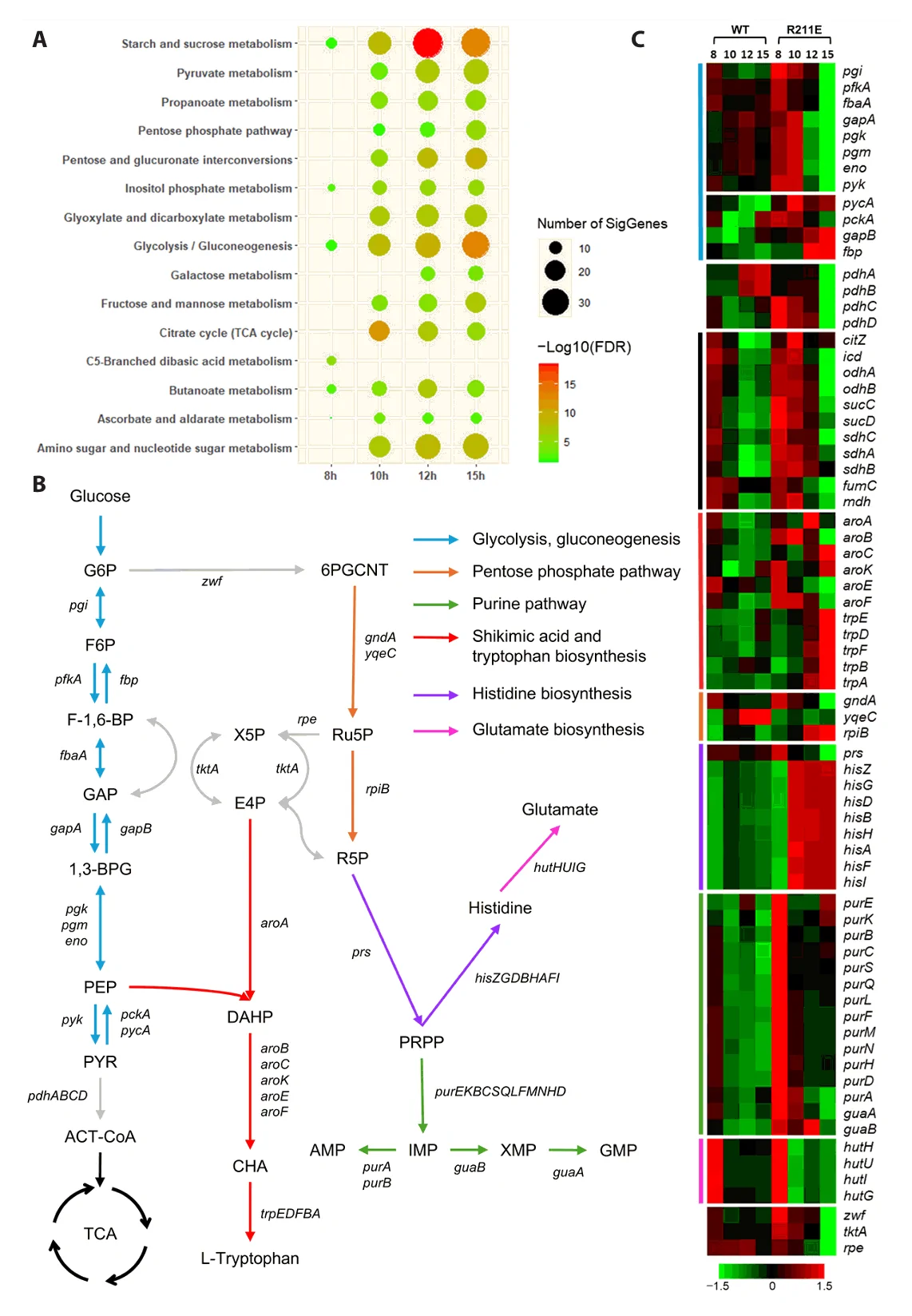
Changes in transcriptome abundance of genes involved in various metabolic pathways. (A) Dotplot of carbohydrate metabolic KEGG pathways. (B) Schematic representation of network connections between glycolysis (blue), pentose phosphate pathway (orange), purine pathway (green), shikimic acid and tryptophan biosynthesis (red), histidine biosynthesis (purple), and glutamate biosynthesis (pink) in *B. subtilis*. The metabolite abbreviations include G6P, glucose 6-phosphate; F6P, fructose 6-phosphate; F-1,6-BP, fructose 1,6-bisphosphate; GAP, glyceraldehyde 3-phosphate; 1,3-BPG, 1,3-bisphosphoglycerate; PEP, phosphoenolpyruvate; PYR, pyruvate; ACT-CoA, acetyl CoA; X5P, xylulose 5-phosphate; E4P, erythrose 4-phosphate; DAHP, 3-deoxy-d-arobino-heptulosonate 7-phosphate; CHA, chorismate; 6PGCNT, 6-phosphogluconate; Ru5P, ribulose 5-phosphate; R5P, ribose-5-phosphate; PRPP, 5-phospho-α-D-ribosyl-1-pyrophosphate; AMP, adenosine 5’-mono-phosphate; IMP, inosine 5’-mono-phosphate; XMP, xanthosine 5’-mono-phosphate; GMP, guanosine 5’-mono-phosphate. (C) Expression patterns of the genes in each pathway are shown as a heatmap, with gene expression visualized as the z-score. The pathways are represented with a color bar corresponding to each in the schematic network. In the heatmap, red and green colors indicate the up-regulated and down-regulated genes, respectively.

**Carbohydrate metabolism:** Pathway analysis indicated changes in the carbohydrate metabolism of R211E (Fig. 6A). DEGs were annotated to 15 carbohydrate metabolism pathways, including starch and sucrose metabolism (bsu00500), glycolysis/gluconeogenesis (bsu00010), amino sugar and nucleotide sugar metabolism (bsu00520), pyruvate metabolism (bsu00030), and the citrate cycle (bsu00020).

*Glycolysis and gluconeogenesis*: The expression levels and fold changes of genes associated with glycolysis and gluconeogenesis are depicted in Fig. 6C. Most genes involved in glycolysis were up-regulated in both strains at exponential growth (8 h and 10 h), with higher expression noted in R211E compared to the WT. Their expression, however, began to decrease from the beginning of stationary phase and dropped significantly in R211E at the middle of stationary phase. In contrast, genes coding for gluconeogenesis such as *pckA* (phosphoenolpyruvate carboxykinase), *gapB* (glyceraldehyde-3-phosphate dehydrogenase), and *fbp* (fructose-1,6-bisphosphatase), exhibited high expression levels in R211E relative to the WT across most time points. Specifically, the expression of *gapB* and *fbp* at the middle of stationary phase was 3.4- and 5.5-fold greater in R211E than in the WT, respectively. These findings indicate that glycolysis and gluconeogenesis are prominent in R211E during the exponential phase (up to 10 h), followed by a reduction in glycolysis after the beginning of stationary phase while gluconeogenesis continues to be active.

*Citrate cycle*: The expression levels and fold changes of genes associated with the citrate cycle are depicted in Fig. 6C. GO analysis revealed that the citrate cycle (GO:0006099) was up-regulated in R211E compared to WT at the end of exponential growth and the beginning of stationary phase. At the middle of exponential growth, genes involved in the cycle, such as *citZ* (citrate synthase), *odhAB* (2-oxoglutarate dehydrogenase), *sucCD* (succinyl-CoA synthetase), and *sdhCAB* (succinate dehydrogenase), were highly expressed in both strains. However, their expression in WT was down-regulated from the end of exponential growth onwards, whereas in R211E, it remained high until the beginning of stationary phase. Interestingly, in the middle of stationary phase, the expression of citrate cycle-related genes in R211E sharply decreased, exhibiting a negative fold change for most genes. These findings suggest that in R211E, the citrate cycle is actively maintained until the beginning of stationary phase but becomes abruptly down-regulated at the middle of stationary phase.

*Pentose phosphate pathway*: Three genes involved in the pentose phosphate (PP) pathway, *gndA* (NADP-dependent 6-P-gluconate dehydrogenase), *yqeC* (putative 6-phosphogluconate dehydrogenase), and *rpiB* (ribose 5-phosphate epimerase), were among the DEGs. Their expression levels and fold changes are depicted in Fig. 6C. The expression patterns of these genes distinctly categorized them into two groups: those that were down-regulated (*gndA* and *yqeC*) and those that were up-regulated (*rpiB*) in R211E. These groups of genes encode enzymes that act before and after ribulose 5-phosphate (Ru5P) in the PP pathway, respectively (Fig. 6B). This regulatory mechanism likely aims to enhance ribose-5-phosphate (R5P) production for histidine and purine synthesis (see sections “Amino acid metabolism” and “Nucleotide metabolism”).

**Amino acid metabolism:** Biosynthesis of several amino acids was up-regulated in R211E compared to WT. The histidine biosynthetic process (GO:0000105) was up-regulated in both strains from the middle to the end of exponential growth. In WT, this process was down-regulated from the end of exponential growth to the beginning of stationary phase. However, this down-regulation did not occur in R211E, resulting in constant up-regulation compared to WT from the end of exponential growth. This is supported by the observed extreme up-regulation of the histidine biosynthesis operon gene (Table 2, Fig. 6C). Similarly, the arginine biosynthetic process (GO:0006526) exhibited sustained up-regulation in R211E compared to WT from the end of exponential growth to the middle of stationary phase. It was down-regulated in WT at the second timepoint (the end of exponential growth to the beginning of stationary phase), while it was up-regulated at the first timepoint in R211E (the middle to the end of exponential growth). The tryptophan biosynthetic process (GO:0000162) was also up-regulated in R211E at the beginning and the middle of stationary phase compared to WT. This term was detected as up-regulated at timepoints from the beginning and the middle of stationary phase in R211E. In R211E, both leucine (GO:0009098) and valine (GO:0009099) biosynthetic processes were up-regulated from the middle to the end of exponential growth. Meanwhile, glutamate metabolic processes (GO:0006536) were down-regulated at the first timepoint (the middle to the end of exponential growth) in both strains. Consistently, the histidine utilization *hut* operon genes involved in glutamate synthesis, were down-regulated from the middle of exponential growth in both strains, with a more pronounced decrease in R211E (Fig. 6C). These observed regulations suggest that there are predictable changes in amino acid metabolism in R211E.

**Nucleotide metabolism:** The nucleotide biosynthetic process (GO:0009117), particularly the purine nucleotide biosynthetic process (GO:0006164), exhibited up-regulation at the middle and end of exponential growth in R211E compared to WT. In the KEGG pathway analysis, purine metabolism (bsu00230) showed enrichment at all time points (R211E/WT, Table S5). As shown in Fig. 6B, the expression patterns of genes associated with the purine pathway were consistent in both strains, WT and R211E, displaying high expression at the middle of exponential growth and reduced expression from the end of exponential growth to the middle of stationary phase. Nevertheless, expression levels were higher in R211E, indicating that purine nucleotide synthesis is more actively pursued particularly during exponential growth, in R211E.

**Fig. 7.**
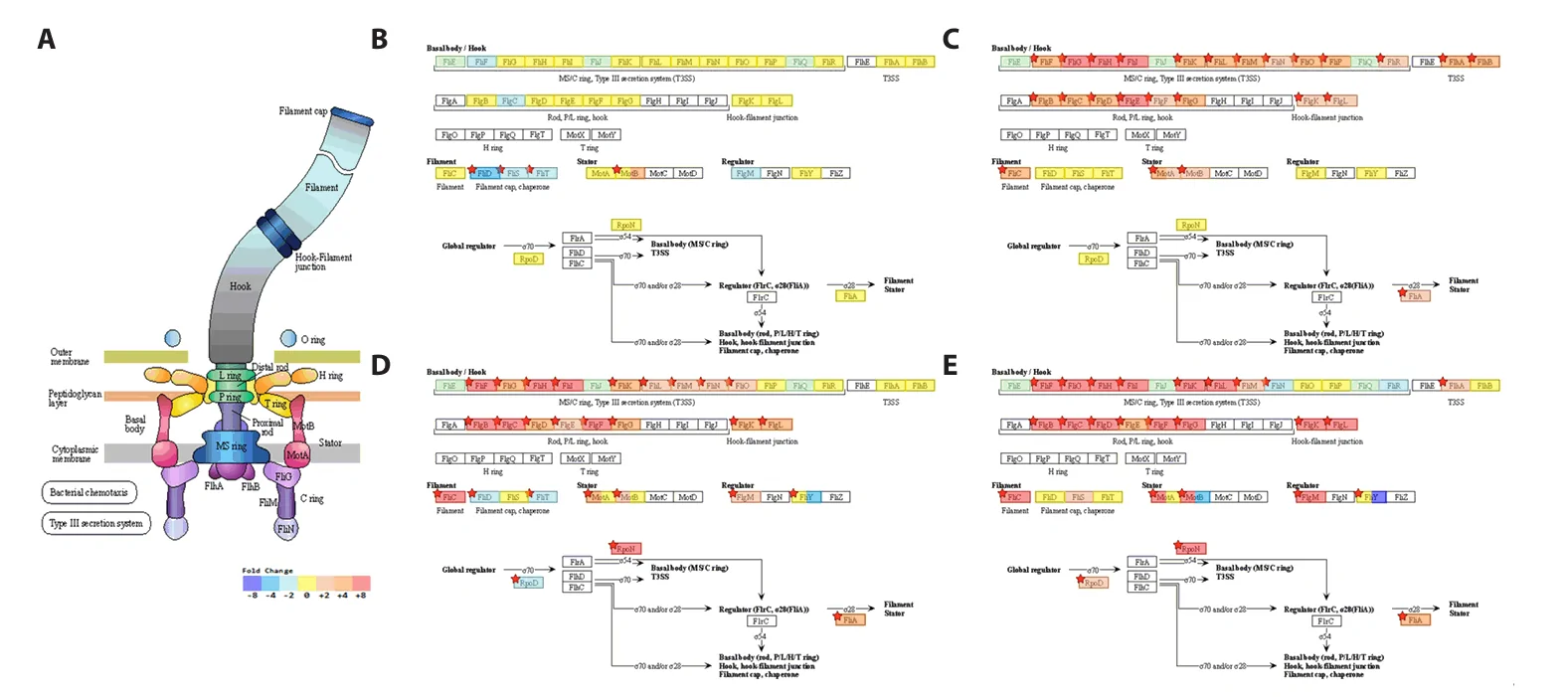
KEGG pathway enrichment of flagellar assembly. (A) Schematic diagram of genes involved in flagellar assembly. Flagellar assembly pathway highlighting the differentially expressed genes in R211E versus WT at (B) the middle of exponential growth (8 h), (C) the end of exponential growth (10 h), (D) the beginning of stationary phase (12 h), and (E) the middle of stationary phase (15 h). Fold change values are expressed using the color key. Blue, red, and yellow colors denote down-regulated, up-regulated, and non-differentially expressed genes, respectively. Green indicates genes that are not mapped, and genes marked with a red star are considered significantly different.

**Flagellar and chemotaxis:** As revealed by the KEGG analysis, at least 26 genes related to the flagellar assembly (bsu02040) pathway were identified at timepoints after the end of exponential growth (Table S5). Similarly, the chemotaxis (GO:0006935) was enriched in up-regulated DEGs of R211E/WT at the end of exponential growth and the middle of stationary phase (Table S4). As shown in Fig. 7, many flagellar-related genes, especially components of the flagellar structure, were significantly up-regulated in R211E after the end of exponential growth. These results indicate that the function loss of Spo0A affects the mobility of this strain.

**Fig. 8.**
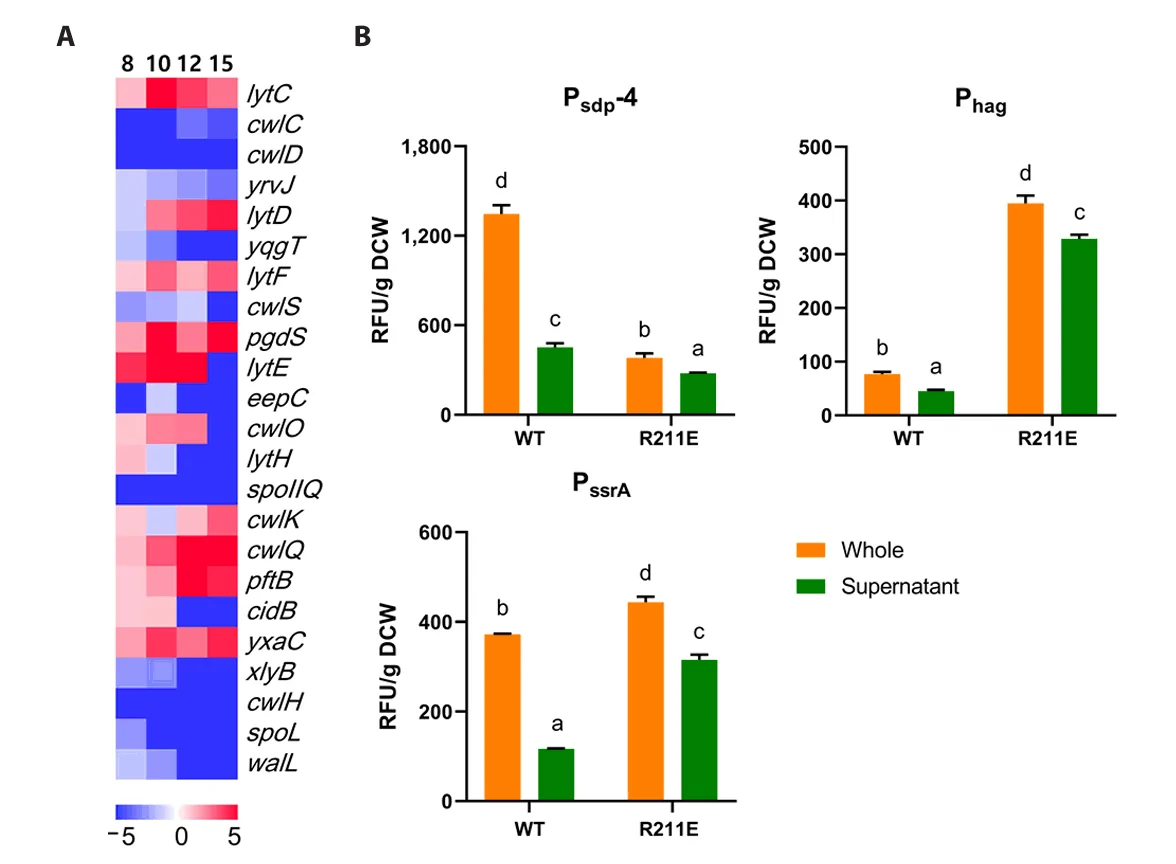
Expression of peptidoglycan hydrolase genes and relative eGFP expression. (A) Heatmap representing the expression of peptidoglycan hydrolase genes (log2 fold change). In the heatmap, red and blue colors indicate the up-regulated and down-regulated genes, respectively. (B) The relative eGFP expression per gram of dry cell weight. Relative fluorescence unit (RFU) values in WT and R211E driven by P_sdp_-4, P_hag_, and P_ssrA_ at 48 h, are expressed in terms of grams of dry cell weight (DCW). The eGFP fluorescence in the whole culture sample is displayed in the orange bar, while that in the supernatant after centrifugation appears in the green bar. All error bars indicate the standard deviation, derived from three independent experiments. Different lowercase letters above the columns represent significant differences (*p* < 0.05, ANOVA followed by Duncan’s post hoc test).

**Cell wall lytic enzymes and autolysis:** The cell wall organization or biogenesis (GO:0071554) exhibited down-regulation in R211E compared to WT at the middle of stationary phase. As illustrated in Fig. 8A, numerous peptidoglycan hydrolase genes, particularly those related to sporulation, such as *cwlD* and *spoIIQ*, were down-regulated in R211E. Conversely, genes of major autolysin during vegetative growth, *lytC*, *lytF*, and *lytE*, were up-regulated in the strain. These findings indicate that the R211E strain may exhibit modified cell wall remodeling and autolysis mechanisms.

### Protein expression in R211E

To investigate the impact of Spo0A disruption on protein expression, the pUB19-P_sdp_-egfp-4 expression vector, which exhibited strong eGFP expression in WT (Jun et al., 2023a), was introduced into R211E. As shown in Fig. 8B, protein expression by P_sdp_-4 expression cassette was significantly decreased in R211E. However, the proportion of total eGFP present in the supernatant was 73% in R211E, while it was 34% in WT. This issue is assumed to occur because the *sdp* operon is positively regulated by Spo0A. Consequently, expression was further examined using the *hag* promoter (P_hag_), which is unaffected by Spo0A. Figure 8B shows that the fluorescence driven by P_hag_ was not reduced, and indeed was higher in R211E, with the proportion of eGFP in the supernatant exceeding that in WT. Additionally, the *ssrA* promoter (P_ssrA_), which showed the highest TPM value in RNA-seq data of R211E, was used for eGFP expression. The P_ssrA_ driven- eGFP protein in the supernatant accounted for 71% in R211E, markedly higher than in WT (31%). These results suggest that the sporulation-deficient mutant R211E has the capability to release recombinant protein into the supernatant.

## Discussion

**Fig. 9.**
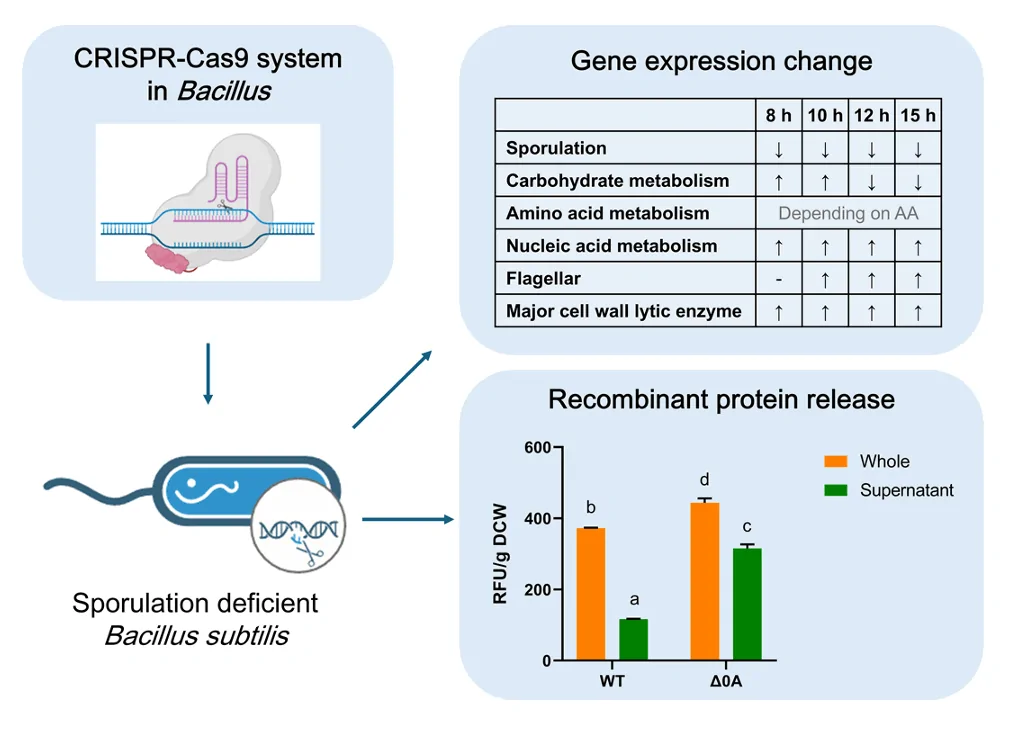
Overview of Transcriptomic Changes in *B. subtilis* Spo0A R211E. This summary figure illustrates the key findings of this study, comparing the R211E mutant and WT across different growth phases. The R211E strain, generated using the CRISPR-Cas9 system, resulted in sporulation deficiency. Transcriptomic analysis revealed loss-function-mutation of Spo0A affecting genes involved in multiple biological processes, including sporulation, carbohydrate metabolism, flagellar assembly, and cell wall lytic enzyme. Finally, we demonstrate the potential application of R211E as a recombinant protein expression system, as it facilitated protein release into the supernatant, offering valuable insights for future metabolic engineering and bioproduction in *B. subtilis*.

In this study, we examined the impact of loss-of function mutation of Spo0A in *B. subtilis* on gene and heterologous protein expression. We previously investigated gene expression changes in *B. subtilis* DB104 during growth (Jun et al., 2023b). Here, the influence of Spo0A mutation in *B. subtilis* DB104 on transcriptome, which results in sporulation deficiency, was assessed using a time-course analysis (Fig. 9).

A CRISPR-Cas9 gene editing system was employed to create Spo0A-mutated strains (R211E). No colonies formed when pUB19-Cas90A was used for transformation. The vector pUB19 originates from the pUB110 plasmid (Kang et al., 2019), which has a high copy number range of 50–100 per cell (Keggins et al., 1978). Consequently, pUB19-Cas90A could induce high levels of Cas9 expression, leading to off-target cleavage, excessive cleavage, and resulting cell death (Zhang et al., 2014). Since CRISPR-Cas9 mutagenesis requires a balance between cleavage and homologous recombination, overexpression of Cas9/gRNA may eliminate cells before repair can occur. Optimizing Cas9/gRNA expression could enhance mutant recovery by allowing more time for recombination with donor DNA. The significance of adjusting Cas9 protein levels has also been documented in *B. thuringiensis* (Soonsanga et al., 2020). To remediate this, a G → T conversion at position 7 of the *repB* gene was implemented, causing an approximately 4-fold reduction in the copy number of pUB110 (Leonhardt, 1990). This adjustment resulted in a low copy number pUB19L-Cas90A, facilitating successful gene editing (Fig. 1B). The structure of Spo0A consists of two functional domains: a phosphorylation domain and a DNA-binding domain, separated by a hinge region (Grimsley et al., 1994). The Arg211 in the Helix-turn-Helix region of the DNA-binding domain, an essential amino acid for the efficient function of Spo0A (Hou et al., 2016; Zhao et al., 2002) was changed from positively charged Arg to negatively charged Glu, disrupting the interaction between Arg and the negatively charged DNA backbone (Zhao et al., 2002). This single amino acid modification effectively abolished sporulation (Fig. 1D). The use of CRISPR-Cas9 eliminates concerns about residual selection markers or unintended mutations, making it particularly suitable for transcriptome studies where genetic background consistency is critical (Tasan and Zhao, 2017; van Leeuwe et al., 2019).

The functional analysis of DEGs indicated significant alteration in transcript levels between R211E and WT across biological processes including sporulation, carbohydrate metabolism, chemotaxis, and autolysis. Since Spo0A serves as a master regulator for initiating sporulation in *B. subtilis* (Marathe et al., 2023), its disruption led to the down-regulation of genes in the downstream of the Spo0A cascade, resulting in a deficiency in sporulation. A key finding from the KEGG pathway analysis was the impact of the Spo0A mutation on numerous genes associated with quorum sensing. This pathway is connected to sporulation, competence, biofilm formation, and degradative enzyme expression (Anju et al., 2018; Kumar and Singh, 2013; Lu et al., 2023; Piazza et al., 1999). Notably, the *comK* gene, crucial for genetic competence in *B. subtilis* (Van Sinderen and Venema, 1994), exhibited consistent down-regulation at all observed time points in our dataset. Consequently, a higher quantity of plasmid was required to transform R211E strain (refer to ‘Materials and Methods’ above). In *B. subtilis*, quorum sensing regulates major physiological processes, including sporulation and competence development. Two key quorum sensing signals, competence and sporulation factor (CSF) and ComX, mediate these processes (Solomon et al., 1995). They exhibit regulatory crosstalk and balance between these two quorum sensing mechanisms determines whether the population prioritizes competence or sporulation, depending on environmental conditions and cell density (Schultz et al., 2009). Given that Spo0A phosphorylation is crucial for responding to CSF signaling (Fujita and Losick, 2005), the loss-function mutation of Spo0A may disrupt this regulatory balance, potentially shifting the transcriptional landscape of quorum-sensing-related genes. Our transcriptomic analysis suggests that ComA-regulated genes are differentially expressed in R211E, indicating a broader impact on quorum sensing networks. Spo0A and AbrB are transcription factors that regulate biofilm formation (Hamon and Lazazzera, 2001). Spo0A functions as a regulator by binding to and subsequently repressing the promoter of *abrB* (Strauch et al., 1990). Since AbrB acts as negative regulator inhibiting the biofilm formation initiation (Hamon et al., 2004), the repression of *abrB* by Spo0A promotes biofilm development by eliminating this inhibitory influence. Consequently, disruption of Spo0A results in inhibited biofilm formation in *B. subtilis*. Hamon and Lazazzera (2001) observed a biofilm defect in the Spo0A mutant as well. Additionally, the flagellum serves as a crucial motor organ, essential for the mobility and chemotaxis of bacterial cells (Colin et al., 2021). Despite reports that flagella are crucial for initiating biofilm formation (Lemon et al., 2007; Watnick et al., 2001), biofilm formation and flagellar motility appear to be inversely regulated in *B. subtilis*. Expression of flagellar basal body genes has been moted to decrease as biofilm formation progresses (Kobayashi, 2007). Furthermore, flagellar filament gene expression is repressed within the biofilm (Vlamakis et al., 2008). Similarly, Benyoussef et al. (2022) reported that motile *E. coli* exhibit disadvantages in biofilm formation. In this study, KEGG pathway analysis revealed that R211E shows up-regulated flagellar gene expression after 10 h, resulting in colonies with larger and more transparent morphology than those of the WT (Fig. 1C).

In addition, R211E exhibited altered metabolic pathways of carbohydrates, amino acids, and nucleotides. The viable cell counts of R211E was higher than that of WT before entering the stationary phase (Fig. 2B). The genes associated with glycolysis and the TCA cycle were up-regulated in the strain from the middle of exponential growth (8 h), suggesting an increased carbohydrate flux to generate cellular energy for growth. A high demand for ATP accelerates the rate of glycolysis (Koebmann et al., 2002), and pyruvate metabolism is crucial for cell division in *B. subtilis* (Monahan et al., 2014). Several amino acids, particularly histidine biosynthesis, were elevated in R211E from the end of exponential growth onward. Furthermore, purine biosynthesis, which shares the intermediate metabolite PRPP with histidine biosynthesis, also increased in R211E at the middle of exponential growth. Possible reasons for these metabolic changes include involvement of histidine biosynthetic enzymes in cellular division (Gibert and Casadesús, 1990) and the association of histidine biosynthesis with metal homeostasis and survival in low pH environments (Beetham et al., 2024; Dietl et al., 2016). Therefore, harsh conditions in late growth may lead to an increase in histidine metabolism.

Another notable observation was that the expression levels of various cell wall lytic enzymes were either up- or down-regulated. As indicated in Fig. 2B, the cell count of R211E sharply declined after entering the stationary phase. A previous study demonstrated that the *spo0A* mutant strain contains a high level of autolysins (Kodama et al., 2007). *B. subtilis* produces several cell wall lytic enzymes during vegetative growth (Yamamoto et al., 2003), such as LytC (Kuroda and Sekiguchi, 1991), LytD (Margot et al., 1994), LytE (Margot et al., 1998), and LytF (Ohnishi et al., 1999), which are major autolysins in this organism. As shown in Fig. 8A, their expression levels were elevated in R211E. Conversely, the expression levels of sporulation-related autolysins, such as CwlC (Kuroda et al., 1993), CwlD (Sekiguchi et al., 1995), LytH (Horsburgh et al., 2003), and SpoⅡQ (Londoño‐Vallejo et al., 1997), were reduced in R211E. The *spo0A* mutant exhibits low extracellular protease activities (Ferrari et al., 1986), and a deficiency in extracellular protease predisposes cells to lysis (Stephenson et al., 1999). Our results suggest that the rapid decline in cell viability of the Spo0A mutant during the stationary phase was due to increased expression of vegetative autolysin.

Based on these results, we hypothesized that the produced recombinant protein could be released into the supernatant when R211E was used for expression. As anticipated, 73% of the total P_sdp_-4 derived-eGFP was released into the supernatant in R211E (Fig. 8B). However, the total amount significantly decreased compared to WT. Therefore, we employed promoters whose expression is unaffected by Spo0A (P_hag_ and P_ssrA_). The up-regulated vegetative autolysin is expected to have contributed to these protein release. However, cell lysis may also lead to cytoplasmic protein leakage, which could complicate purification. These results suggest that R211E facilitates protein release into the supernatant, offering a potential advantage for recombinant protein production. While further validation is needed to optimize protein yield, and purification efficiency, strategic promoter selection and cell lysis could enhance its applicability in protein expression system. Future studies exploring these factors may help establish R211E as an efficient host for recombinant protein expression.

In conclusion, our transcriptome analysis study identified changes in genetic expression in *B. subtilis* DB104 following the disruption of Spo0A. We created the Spo0A mutated strain, R211E, using the CRISPR-Cas9 system, demonstrating that the optimal amount of Cas9 is crucial for gene editing in *B. subtilis*. Functional analysis of DEGs indicated that an increase in the vegetative cell number of R211E was due to up-regulation in carbohydrate, amino acid, and nucleotide metabolism. Additionally, increased expression of vegetative autolysin prompted a rapid decrease in the cell density of R211E. Our data also revealed a potential application of R211E as a recombinant protein expression system, with efficient protein release into the supernatant. This study provides valuable insights for further metabolic engineering and the development of recombinant expression systems.

## Figures and Tables

**Table 1.** Bacterial strains and plasmids used in this study

Strains and plasmids	Description	Resource
**Strains**		
*E. coli* DH5α	F^-^ Φ80*lac*ZΔM15 Δ(*lac*ZYA-*arg*F) U169 deoR *rec*A1 *end*A1 *hsd*R17 (rK^-^, mK^+^) *pho*A *sup*E44 λ^-^ *thi*-1 *gyr*A96 *rel*A1	Hanahan (1983)
*B. subtilis*		
DB104 (WT)	*his npr*R2* npr*E18 Δ*aprA3*	Kawamura and Doi (1984)
DB104 Spo0A R211E (R211E)	DB104 with mutation of *spo0A* R211E (CGT → GAA)	This study
**Plasmids**		
pUB19	*E. coli*-*Bacillus* shuttle vector, Ap^r^, Km^r^	Kang et al. (2019)
pCas9	Bacterial expression of Cas9 nuclease, tracrRNA and crRNA guide, pACYC184 backbone, Cm^r^	addgene#42876
pP_amyE_-Cas9	Derivative of pCas9, replaced with P_amyE_	This study
pP_amyE_-Cas90A	Derivative of pP_amyE_ -Cas9, includes 20 bp sgRNA targeting *spo0A*	This study
pUB19-Cas90A	Derivative of pP_amyE_ -Cas90A; includes tracrRNA, Cas9, and crRNA; 20 bp sgRNA targeting *spo0A*	This study
pUB19L-Cas90A	pUB19-Cas90A derivative, converted to a low-copy-number origin (*repB*)	This study
pUC19	*E. coli* vector, Ap^r^	Lab collection
pUC19-R0A	pUC19 derivative, containing 430 bp donor DNA for generating *spo0A* mutation	This study
pUB19-P_sdp_-egfp-2	P_sdp_-2 expression cassette, eGFP, Ap^r^, Km^r^	Jun et al. (2023a)
pUB19-P_sdp_-egfp-4	P_sdp_-4 expression cassette, eGFP, Ap^r^, Km^r^	Jun et al. (2023a)
pUB19-P_hag_-egfp	*hag* promoter, eGFP, Ap^r^, Km^r^	This study
pUB19-P_ssrA_-egfp	*ssrA* promoter, eGFP, Ap^r^, Km^r^	This study

Ap: Ampicillin, Km: Kanamycin, Cm: Chloramphenicol,

r : resistance

**Table 2.** The top 10 most up- and down-regulated DEGs according to fold change

Locus tag	Gene	Description	Fold change	Adj. *p*-val^a^	Comparison type
BSU_34870	*hisF*	Imidazole glycerol phosphate synthase subunit	3220.74	1.22 × 10 ^−205^	R211E, 10 h / 8 h
BSU_34920	*hisG*	ATP phosphoribosyltransferase	1823.43	1.65 × 10 ^−175^	R211E, 10 h / 8 h
BSU_34900	*hisB*	Imidazoleglycerol-phosphate dehydratase	1319.59	1.38 × 10 ^−176^	R211E, 10 h / 8 h
BSU_34860	*hisI*	Bifunctional phosphoribosyl-AMP cyclohydrolase	1313.22	3.48 × 10 ^−203^	R211E, 10 h / 8 h
BSU_34890	*hisH*	Imidazole glycerol phosphate synthase	1289.17	2.12 × 10 ^−186^	R211E, 10 h / 8 h
BSU_34910	*hisD*	Histidinol dehydrogenase	1103.91	5.63 × 10 ^−191^	R211E, 10 h / 8 h
BSU_34880	*hisA*	Phosphoribosylformimino-5-aminoimidazole carboxamide ribotide isomerase	948.15	1.81 × 10 ^−173^	R211E, 10 h / 8 h
BSU_34930	*hisZ*	Histidyl-tRNA synthetase	922.49	5.68 × 10 ^−169^	R211E, 10 h / 8 h
BSU_17450	*glnR*	Transcriptional regulator	536.98	2.03 × 10 ^−41^	R211E/WT, 15 h
BSU_22200	*cotD*	Spore coat protein	291.36	1.27 × 10 ^−41^	WT, 15 h / 12 h
BSU_11750	*cotY*	Outer spore coat protein	−137476.94	8.41 × 10 ^−27^	R211E/WT, 15 h
BSU_11740	*cotZ*	Spore coat protein	−90494.67	6.03 × 10 ^−26^	R211E/WT, 15 h
BSU_09750	*sspB*	Small acid-soluble spore protein	−30569.08	5.17 × 10 ^−24^	R211E/WT, 15 h
BSU_09750	*sspB*	Small acid-soluble spore protein	−17694.34	1.38 × 10 ^−30^	R211E/WT, 15 h
BSU_06910	*cotJC*	Enzyme component of the inner spore coat	−16731.66	3.80 × 10 ^−42^	R211E/WT, 15 h
BSU_40530	*cotF*	Spore coat protein	−14976.10	2.11 × 10 ^−20^	R211E/WT, 15 h
BSU_misc_RNA_86	*srlX*	Putative small RNA or mRNA leader sequence	−10554.53	1.14 × 10 ^−14^	R211E/WT, 8 h
BSU_39890	*yxbB*	Putative S-adenosylmethionine-dependent methyltransferase	−7381.12	1.23 × 10 ^−11^	R211E/WT, 8 h
BSU_11760	*cotX*	Spore coat protein	−7204.75	2.76 × 10 ^−25^	R211E/WT, 15 h
BSU_28410	*gerE*	Transcriptional regulator of SigK-dependent late spore coat genes	−5941.51	6.68 × 10 ^−21^	R211E/WT, 15 h

a *p*-value after FDR correction.

## Data Availability

The datasets supporting the conclusions of this article are included within the article as well as their additional files. The raw sequence data have been submitted to the Sequence Read Archive (SRA, https://www.ncbi.nlm.nih.gov/sra) under Bioproject accession number PRJNA1162568 and reference Biosample accession number SAMN43816647—50.

## Supplementary Information

Table S1. Oligonucleotides used in this study

Table S2. Summary of RNA-seq and the reads mapped to the genome of *Bacillus subtilis* subsp. subtilis str. 168

Table S3. Primers used for RT-qPCR analysis

Table S4. Enriched GO terms in biological process

Table S5. Enriched KEGG pathways

Fig. S1. Construction of the pUB19L-Cas90A. (A) Construction scheme for the pUB19L-Cas90A. (B) Design of a *spo0A* targeting sgRNA.

Fig. S2. Construction of the pUC19-R0A.

Fig. S3. Volcano plots of differentially expressed genes (DEGs) showing the fold change vs. the *p*-value in (A) WT (10 h versus 8 h, 12 h versus 10 h, and 15 h versus 12 h), (B) R211E (10 h versus 8 h, 12 h versus 10 h, and 15 h versus 12 h), and (C) R211E versus WT (8 h, 10 h, 12 h, and 15 h). Yellow and blue dots indicate up- and down-regulated DEGs with significant differences satisfying the DEG rules, respectively (*p*-value < 0.05, |fold change| ≥ 2). Gray dots indicate non-differentially expressed genes.

Fig. S4. Real-Time quantitative PCR (RT-qPCR) validation of RNA-seq data for selected genes with top fold changes. The left and right y-axes represent the TPM value of RNA-seq and the fold change of RT-qPCR, respectively. Fold-change values for RT-qPCR samples were determined by comparing gene expression at each time point against that at 8 h. RT-qPCR analyses of RNA expression of target genes were normalized to *rpsJ*. RT-qPCR data are presented as mean ± standard deviation (n = 3).
