# Supplementary material for: Time-resolved analysis of Bacillus subtilis DB104 Spo0A-mutant transcriptome profile and enhancement of recombinant protein release
Source: J Microbiol. 2025 May 27;63(5):e2411032. doi: 10.71150/jm.2411032 (PMC13577027; doi:10.71150/jm.2411032)
Supplement: Supplementary file 1 [file jm-2411032-Supplementary-Fig-S1.pdf]

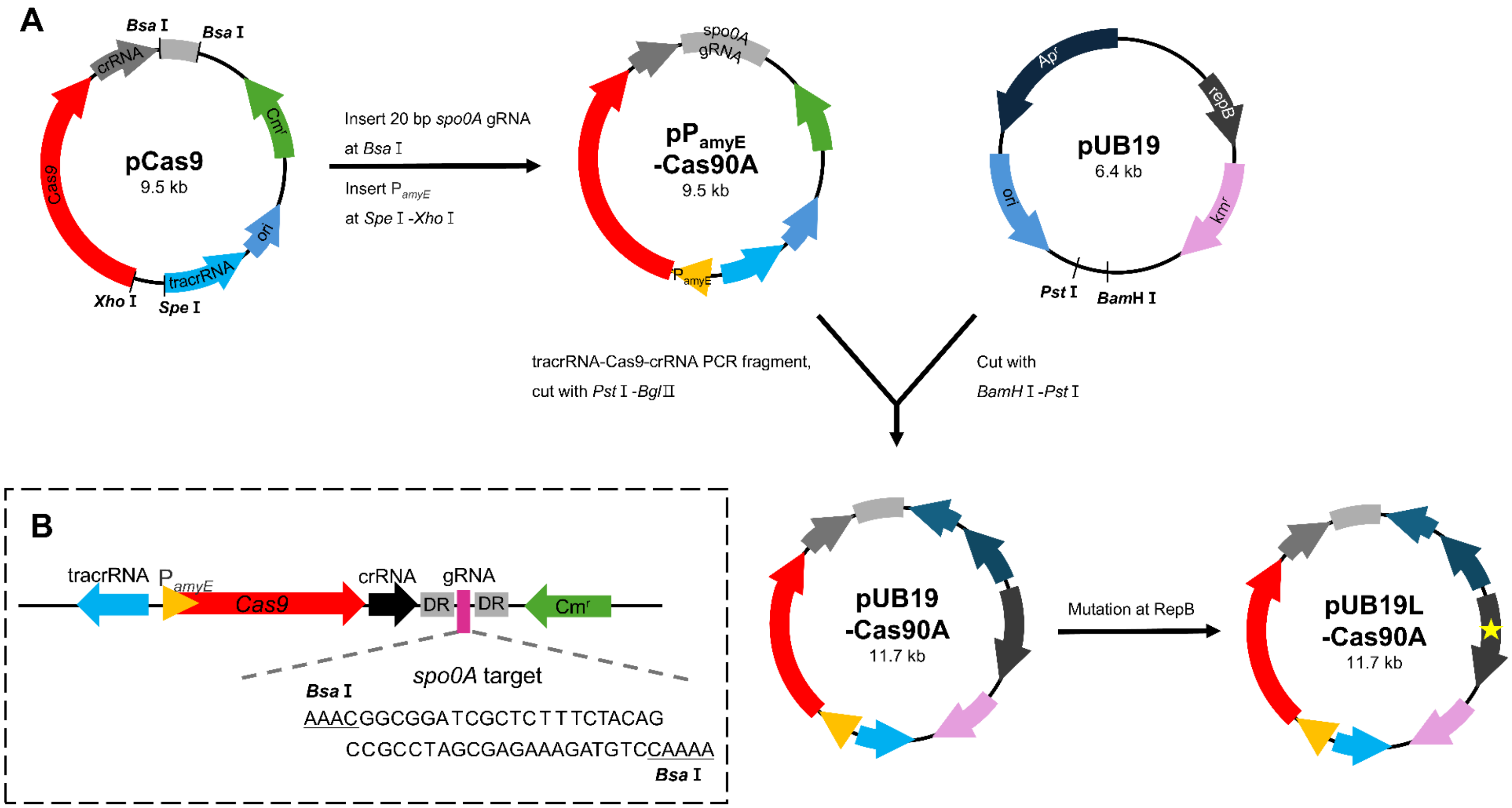

Fig. S1. Construction of the pUB19L-Cas90A. (A) Construction scheme for the pUB19L-Cas90A. (B) Design of a *spo0A* targeting sgRNA.
