# Supplementary material for: Time-resolved analysis of Bacillus subtilis DB104 Spo0A-mutant transcriptome profile and enhancement of recombinant protein release
Source: J Microbiol. 2025 May 27;63(5):e2411032. doi: 10.71150/jm.2411032 (PMC13577027; doi:10.71150/jm.2411032)
Supplement: Supplementary file 2 [file jm-2411032-Supplementary-Fig-S2.pdf]

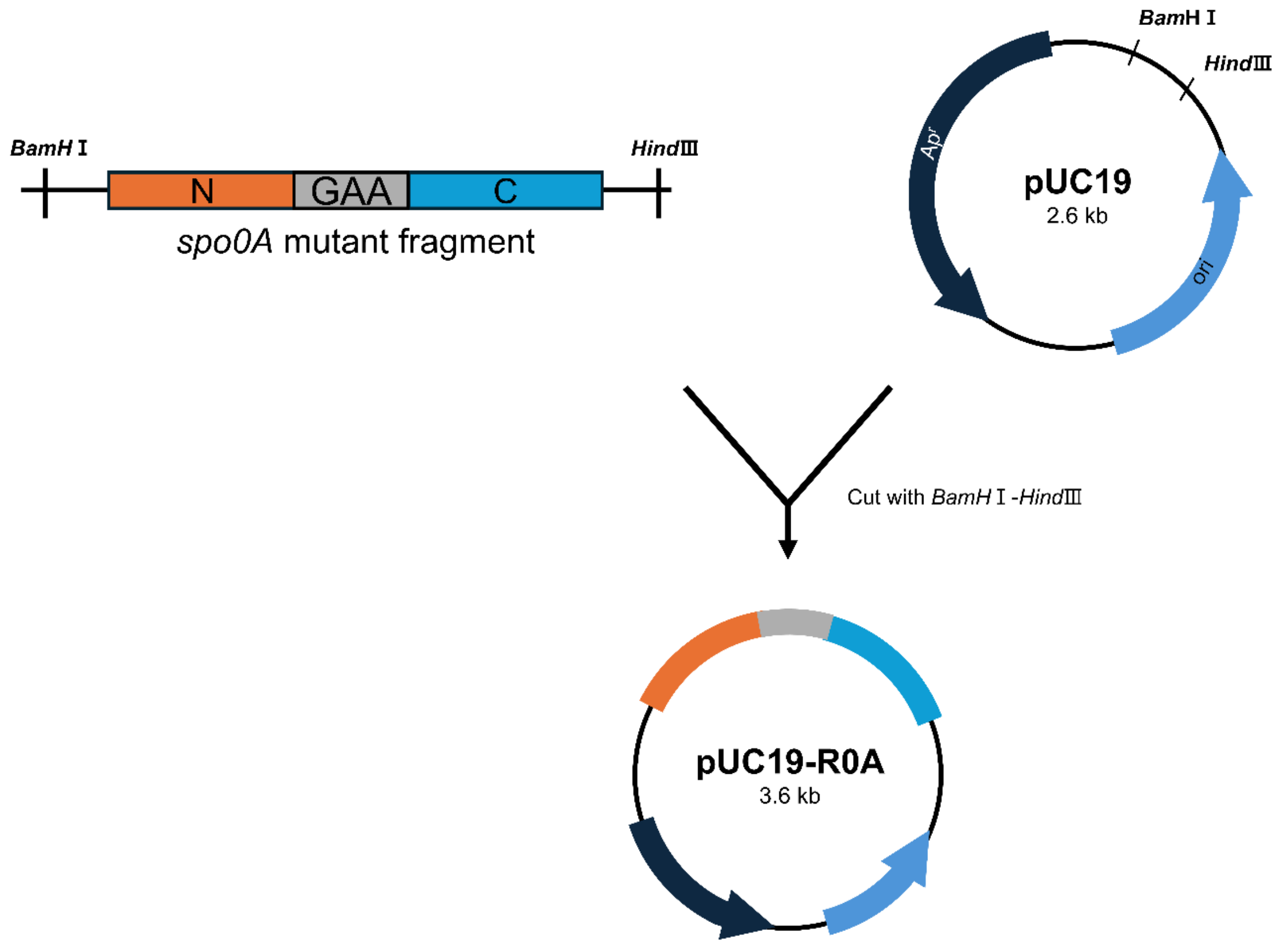

Fig. S2. Construction of the pUC19-R0A.
