# Supplementary material for: Time-resolved analysis of Bacillus subtilis DB104 Spo0A-mutant transcriptome profile and enhancement of recombinant protein release
Source: J Microbiol. 2025 May 27;63(5):e2411032. doi: 10.71150/jm.2411032 (PMC13577027; doi:10.71150/jm.2411032)
Supplement: Supplementary file 3 [file jm-2411032-Supplementary-Fig-S3.pdf]

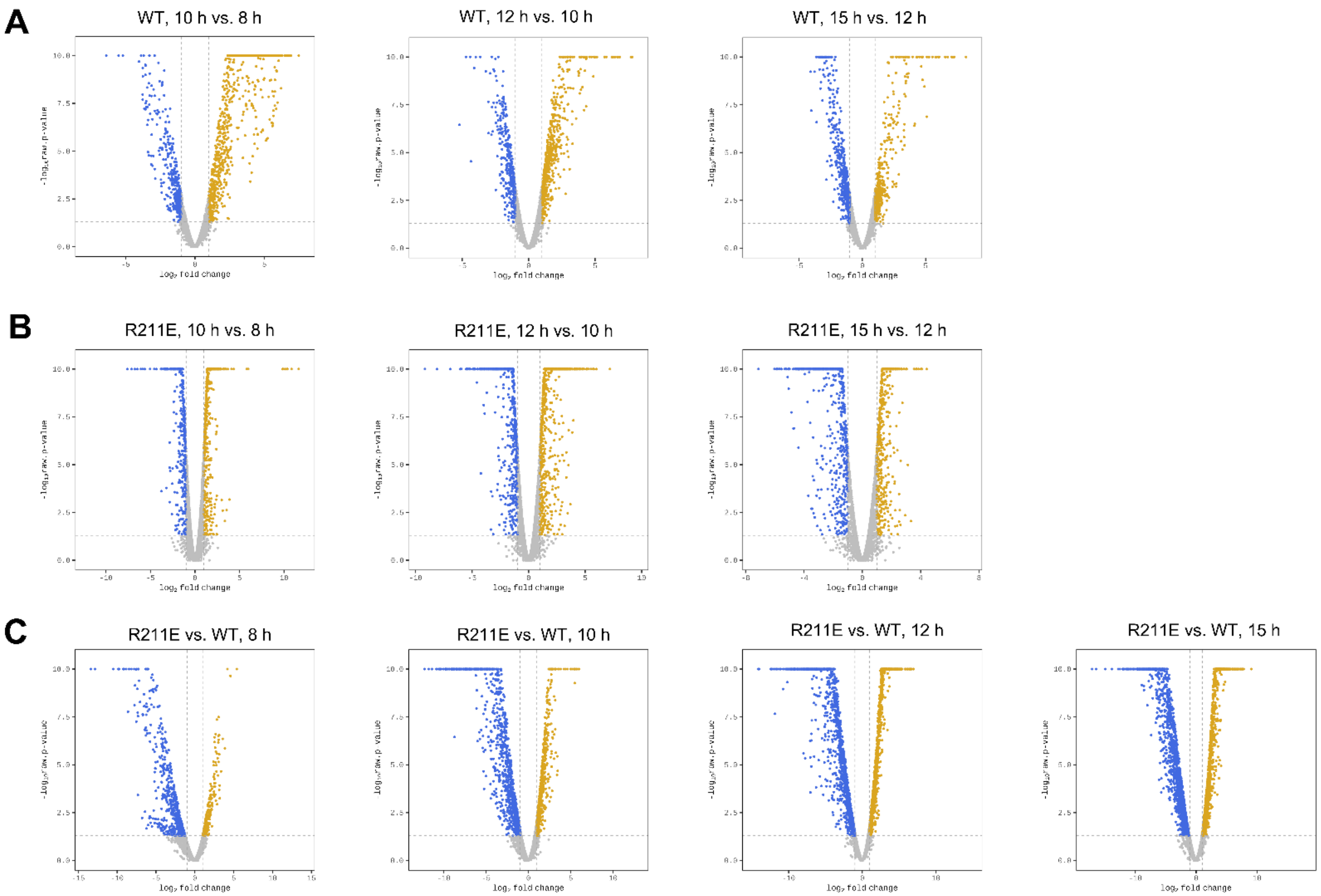

Fig. S3. Volcano plots of differentially expressed genes (DEGs) showing the fold change vs. the p-value in (A) WT (10 h versus 8 h, 12 h versus 10 h, and 15 h versus 12 h), (B) R211E (10 h versus 8 h, 12 h versus 10 h, and 15 h versus 12 h), and (C) R211E versus WT (8 h, 10 h, 12 h, and 15 h). Yellow and blue dots indicate up- and down-regulated DEGs with significant differences satisfying the DEG rules, respectively (p-value < 0.05, |fold change| ≥ 2). Gray dots indicate non-differentially expressed genes.
