# Supplementary material for: Time-resolved analysis of Bacillus subtilis DB104 Spo0A-mutant transcriptome profile and enhancement of recombinant protein release
Source: J Microbiol. 2025 May 27;63(5):e2411032. doi: 10.71150/jm.2411032 (PMC13577027; doi:10.71150/jm.2411032)
Supplement: Supplementary file 4 [file jm-2411032-Supplementary-Fig-S4.pdf]

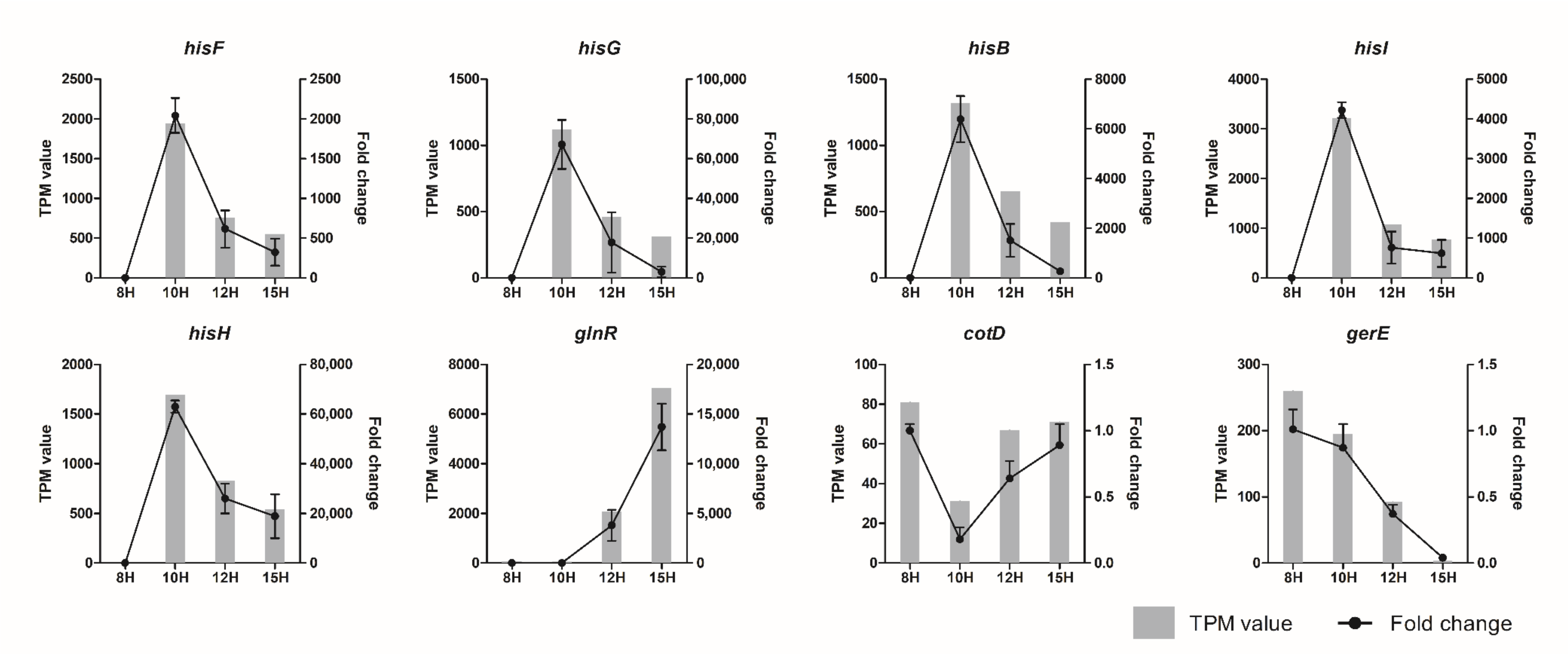

Fig. S4. Real-Time quantitative PCR (RT-qPCR) validation of RNA-seq data for selected genes with top fold changes. The left and right y-axes represent the TPM value of RNA-seq and the fold change of RT-qPCR, respectively. Fold-change values for RT-qPCR samples were determined by comparing gene expression at each time point against that at 8 h. RT-qPCR analyses of RNA expression of target genes were normalized to *rpsJ*. RT-qPCR data are presented as mean ± standard deviation (n = 3).
